# RETRACTION: ET-1 Promotes Differentiation of Periodontal Ligament Stem Cells Into Osteoblasts Through ETR, MAPK, and Wnt/β-Catenin Signaling Pathways Under Inflammatory Microenvironment

**DOI:** 10.1155/mi/9763858

**Published:** 2025-11-07

**Authors:** Mediators of Inflammation

RETRACTION: L. Liang, W. Zhou, N. Yang, J. Yu, and H. Liu, “ET-1 Promotes Differentiation of Periodontal Ligament Stem Cells Into Osteoblasts Through ETR, MAPK, and Wnt/β-Catenin Signaling Pathways Under Inflammatory Microenvironment,” *Mediators of Inflammation* 2016 (2016): 8467849, https://doi.org/10.1155/2016/8467849.

The above article, published online on 13 January 2016 in Wiley Online Library (wileyonlinelibrary.com), has been retracted by agreement between the journal Chief Editor, Anshu Agrawal, and John Wiley & Sons Ltd. The retraction has been agreed after an investigation into figure concerns initially raised by *Aneurus inconstans* on PubPeer [1].

Specifically:

- Multiple instances of duplicated bands across Figures 2e, 3d, 4b, 5e and 6b;
- Unexpected similarities between bands published in multiple other papers [2–7].

Several attempts by the journal to contact the authors have not resulted in a reply. An assessment by the Chief Editor has determined that the results reported in this paper cannot be considered reliable and it is therefore retracted.
